# Quantitative Aspect of* Leucophyllum frutescens* Fraction before and after Encapsulation in Polymeric Nanoparticles

**DOI:** 10.1155/2017/9086467

**Published:** 2017-11-22

**Authors:** Claudia Janeth Martínez-Rivas, Rocío Álvarez-Román, Catalina Rivas-Morales, Abdelhamid Elaissari, Hatem Fessi, Sergio Arturo Galindo-Rodríguez

**Affiliations:** ^1^Facultad de Ciencias Biológicas, Laboratorio de Nanotecnología, Universidad Autónoma de Nuevo León, Av. Pedro de Alba s/n, 66455 San Nicolás de los Garza, NL, Mexico; ^2^Univ Lyon, Université Claude Bernard Lyon 1, CNRS, LAGEP-UMR 5007, 69622 Lyon, France; ^3^Facultad de Medicina, Departamento de Química Analítica, Universidad Autónoma de Nuevo León, Av. Fco. I. Madero y Dr. E. Aguirre Pequeño s/n, 64460 Monterrey, NL, Mexico

## Abstract

The interest on plants has been focalized due to their biological activities. Extracts or fractions from plants in biodegradable polymeric nanoparticles (NP) provide many advantages on application studies. The encapsulation of the extract or fraction in NP is determined for the establishment of the test dose. HPLC method is an alternative to calculate this parameter. An analytical method based on HPLC for quantification of a hexane fraction from* L. frutescens* was developed and validated according to ICH. Different concentrations of the hexane fraction from leaves (HFL) were prepared (100–600 *μ*g/mL). Linearity, limit of detection, limit of quantification, and intra- and interday precision parameters were determined. HFL was encapsulated by nanoprecipitation technique and analyzed by HPLC for quantitative aspect. The method was linear and precise for the quantification of the HFL components. NP size was 190 nm with homogeneous size distribution. Through validation method, it was determined that the encapsulation of components (1), (2), (3), and (4) was 44, 74, 86, and 97%, respectively. A simple, repeatable, and reproducible methodology was developed for the propose of quantifying the components of a vegetable material loaded in NP, using as a model the hexane fraction of* L. frutescens* leaves.

## 1. Introduction

Since prehistoric times, medicinal plants have been used as treatments for numerous human diseases [1, 2]. Recently, research articles have proved biological activities of some plant extracts as antioxidant [3, 4], anti-inflammatory [4], antimicrobial [5, 6], and anticancer [7] activity. Organic solvents are used in the extraction process to obtain the active plant material [8] and as carrier in the assays for extract application. However, for* in vivo* administration, an organic solvent would result in toxicity for the organism [9].

Different carriers as nanocomposite films [10], microparticles [11], and nanoparticles [12, 13] have been a good alternative as drug delivery systems. Particularly, polymeric nanoparticles (NP) are promising as carriers which present sustained release and protection to the active [14] and targeting to specific organs [15, 16]. During NP preparation the organic solvent has to be eliminated. Researches in natural products encapsulation have demonstrated beneficial effects.* Arrabidaea chica *is a plant with healing properties employed in folk medicine for wound healing, inflammation, and gastrointestinal colic. Servat-Medina et al. studied the antiulcerogenic activity.* A. chica *hydroalcoholic extract was incorporated in chitosan-sodium tripolyphosphate NP. An* in vitro *study in human skin fibroblasts showed biocompatibility.* In vivo *study proved that* A. chica *hydroalcoholic extract-loaded NP enhances its antiulcerogenic activity [17]. Similar behaviors were obtained by Kwon et al. They investigated different activities of the aqueous extract of* Centella asiatica* loaded gelatin NP. Encapsulated aqueous extract presented lower toxicity in human skin fibroblasts. Extract-loaded NP displayed a stronger inhibition of enzymatic activity that affects dermal tissues, flux through mouse skin, and retention. These results showed the potential use of aqueous extract of* C. asiatica* loaded NP in cosmetic industry [18].

As well as drug encapsulation, extract encapsulation involves the NP characterization, loading (%  *L*), and encapsulation efficiency (% EE). Besides, being two of the main parameters determined for NP characterization [19], in* in vitro *and* in vivo *studies, it is important to establish the test dose in treatments. UV-Vis spectrophotometry has been used for extract-loaded NP quantification [17, 20]. However, quantification of low concentrations is limited for its poor sensitivity. High performance liquid chromatography (HPLC) is a method used to separate the marker compounds in extracts or fractions for their subsequent quantification [21, 22]. Sangthong and Weerapreeyakul identified two compounds (sulforaphene and sulforaphane) in* Raphanus sativus* L. var.* caudatus *Alef extracts. Quantification of both molecules was carried out through a validated HPLC method [23]. Thus, development and validation of a method based on separation of known molecules in the plant and their quantification in NP are promising.

However, there are plants less studied as* Leucophyllum frutescens*, Scrophulariaceae family, is commonly known as “cenizo.” This plant is the bush par excellence of Nuevo León, while, in Texas, it is called the “bush barometer” due to its surprising flowering depending on the humidity in the environment and precipitation rain [24].* L. frutescens *has received attention for its anti-*M. tuberculosis *activity. Methanol extract from leaves has presented biological activity against multidrug-resistant (MDR)* M. tuberculosis *strains [25] and two new active compounds from hexane fraction of* L. frutescens *against these bacteria have been described [26, 27]. We posed hexane fraction loaded NP as promising agent against multidrug-resistant (MDR) strain and NP administration in systems involves knowing the fraction encapsulation. In this context, the aim of this study was to propose the development and validation of an analytical method by HPLC using internal marker compounds for the quantification of the hexane fraction from* L. frutescens *leaves (HFL) loaded in NP.

## 2. Materials and Methods

### 2.1. Plant Material and Reagents

*L. frutescens* was collected in Monterrey; N. L. Methanol (Tedia, USA), formic acid (purity: 90%, Millipore, USA), and acetonitrile (J.T. Baker, USA) were HPLC-grade. Purified water was from a Milli-Q water-purification system (Millipore, USA). Poly-L-lactide acid (PLA) (PURASORB, PURAC biochem BV, Gorinchem, HOL) as the NP-formed polymer and polyvinyl alcohol (PVAL, Clariant, Mexico) as stabilizer. Other solvents used were of analytical grade.

### 2.2. Chromatographic Analysis

High performance liquid chromatography assay was performed with a photodiode array detector (HPLC-DAD) (Varian 9065, 9012, ProStar 410, USA). A Synergi™ 4 *μ*m Fusion-RP 80 Å (150 mm × 2.0 mm × 4 *μ*m) column was used with a flow rate of 0.2 mL/min and maintained at 30°C. Two methods were carried out; for method 1, the mobile phase was formic acid 0.1% v/v (A) and methanol (B). Gradient conditions were as follows: a gradual change from 45 : 55 (A : B) to 100 (B) was completed during 0–20 min, and then 100 (B) was maintained for 20–35 min. For method 2, the mobile phase was an isocratic elution with 45 : 55 (A : B) during 40 min. Detection wavelength was set at 210, 215, 220, and 229 nm. The peaks at 210 nm were detected, based on peak areas at the maximum wavelength.

### 2.3. Preparation of Calibration Curve

The leaves of* L. frutescens *(50 g) were dried at room temperature, pulverized, and extracted with methanol (350 mL) with sonication (Ultrasonic Cleaners, VWR Symphony, USA). The methanol extract was evaporated under reduced pressure (Laborota 4003 control, Heidolph). Liquid-liquid partition was carried out with hexane. The hexane fraction from leaves (HFL) was evaporated under reduced pressure. For the preparation of stock solution, the total semisolid HFL was weighed and dissolved in methanol. Then, this solution was filtered through a 0.45 *μ*m membrane filter (Millipore, USA). The working solutions were prepared in a concentration range from 100 to 600 *μ*g/mL in triplicate and filtered for their HPLC-DAD analysis to obtain the calibration curve.

### 2.4. Method Validation

The method was validated for linearity, limit of detection (LOD), limit of quantification (LOQ), and intra- and interday precision according to International Conference on Harmonisation [28]. For the establishment of linearity, five levels of concentration were prepared in a range from 100 to 600 *μ*g/mL in triplicate, while the LOD and LOQ were calculated from the calibration curve according to

(1) LOD=3.3σS,LOQ=10σS,

where *σ* is the standard deviation of the response and *S* is the slope of the calibration curve. The residual standard deviation of a line regression or the standard deviation of *y*-intercepts of lines regression may be used as the standard deviation. Finally, the intra- and interday precision were determined analyzing three concentrations with six replicates each one, during a single day and on three different days, respectively.

Once the chromatographic method was established and validated, NP were prepared according to the procedure of nanoprecipitation technique developed by Fessi et al. [29]. Briefly, the organic phase was prepared by dissolving 30 mg of PLA and 3 mg of hexane fraction in 3 mL of an organic solvent mixture (acetone : methanol). The organic solution was added to 10 mL of aqueous phase containing PVAL (1%, w/w) and stirred magnetically. The organic solvent was evaporated at reduced pressure. NP characterization was carried out determining size and size distribution index (PDI) by dynamic light scattering (DLS) (Zetasizer Nano ZS90, Malvern Instruments, UK). The morphology of the NP was observed using a FEI Quanta 250 FEG Microscope at the* “Centre Technologique des Microstructures”* (CT*μ*, Claude Bernard University Lyon 1, France). For the preparation of SEM samples, a drop of diluted aqueous suspension was deposited on a flat metallic holder and dried at room temperature. The sample was finally coated under vacuum by cathodic sputtering with platinum. The samples were observed by SEM under an accelerating voltage of 10 kV. For encapsulation loading (%  *L*) and encapsulation efficiency (% EE), NP dispersions were centrifuged at 25,000 rpm (Allegra 64R, Beckman Coulter, USA) and obtained pellets were lyophilized (Freeze Dry System, LABCONCO, USA). Lyophilized NP were dissolved in acetonitrile-methanol. Solutions were analyzed by HPLC to quantify the encapsulated peaks by the parameters: %  *L* and % EE, according to the following:

(2) %  L=Amount  of  peak/component  in  NPMass  of  lyophilized  NP×100,%  EE=Amount  of  peak/component  in  NPTotal  amount  of  peak/component×100.

## 3. Results and Discussion

### 3.1. Development of the Chromatographic Method by HPLC

In medicinal plants, there are hundreds of unknown components. Variability within the same herbal materials [21] depends on the collection station and the origin of the plant, among other factors [30]. For this reason, the determination of the chromatographic profile is a useful tool for the quality control of plant extract samples [31–34]. HPLC analysis is carried out for knowledge of the chromatographic profile of the vegetal samples [21].

**Figure 1:**
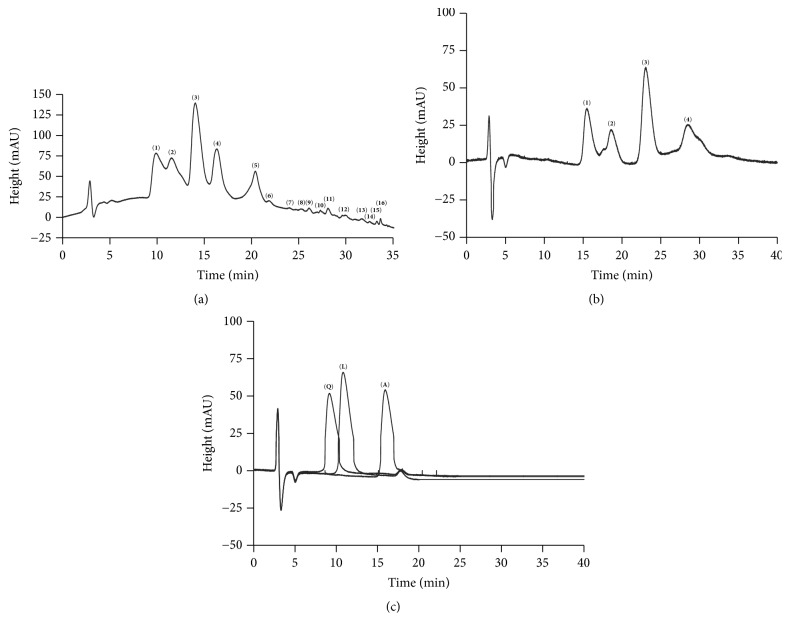
Chromatographic profile of hexane fraction from* L. frutescens* leaves (400 *μ*g/mL) by HPLC (a) method 1: chromatogram shows 16 peaks-components with a retention time of 9.88, 11.50, 14.04, 16.34, 20.40, 21.88, 24.04, 25.30, 26.11, 27.32, 28.11, 29.94, 31.62, 32.52, 33.27, and 33.66 min. (b) Method 2: chromatogram shows 4 peaks-components with a retention time of 15.48, 18.61, 23.05, and 28.49 min. (c) Quercetin (Q), luteolin (L), and apigenin (A) with retention time of 9.31, 10.97, and 16.07 min, respectively, analyzed by method 2.

*L. frutescens *is a plant less studied that has demonstrated to be promising for its use against tuberculosis. In this way, to obtain the chromatographic profile using the hexane fraction of* L. frutescens* leaves increases the knowledge about the plant. Method 1 was developed to obtain the chromatographic profile HFL.  Figure 1(a) shows 16 peaks at a concentration of 400 *μ*g/mL of the fraction and the main peaks are (1)–(4). Method 2 was developed to separate these four main peaks and validated for quantification of peaks (Figure 1(b)). In both chromatograms each peak corresponds to one component of the fraction [35]. Method 2 was validated to quantify the components in the hexane fraction loaded NP.

To increase the knowledge about the plant, a searching for some compounds found in* L. frutescens *family called Scrophulariaceae was performed; three compounds, quercetin [36], luteolin, and apigenin [37], were selected and analyzed by HPLC method 2. The HPLC analysis was carried out and retention time of each one was obtained (Figure 1(c)). Compared with the retention time of the four components in HFL observed by the same method, peak-component (1) (15.48 min) would be apigenin (16.07 min). It is necessary to analyze HFL by spectroscopic methods to assign the presence of this compound.

### 3.2. Validation of Analytical Procedure: Linearity, Limit of Detection, Limit of Quantification, and Intra- and Interday Precision

**Figure 2:**
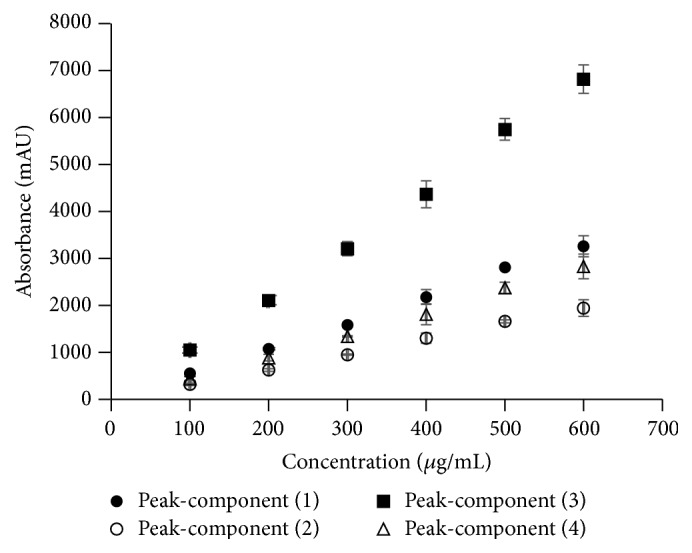
Calibration curve for the four peaks-components present in the hexane fraction from* L. frutescens *leaves (Mean ± SD, *n* = 3).

The developed HPLC-DAD method has demonstrated to be simple, sensitive, specific, and adequate for the simultaneous quantification [38]. The validation was performed to know the linearity and the precision of the chromatographic method (method 2) for the quantification of the peaks-components of HFL loaded NP. For the establishment of linearity, a minimum of 5 concentrations is recommended by the ICH [28]. A calibration curve of the semisolid HFL in acetonitrile-methanol at a concentration range from 100 to 600 *μ*g/mL was prepared. The area under the curve in each concentration level of each peak-component was analyzed to obtain Figure 2. Consequently, regression equation, correlation coefficient, LOD, and LOQ of each peak-component were established (Table 1). The acceptance criterion for linearity is given by the correlation coefficient [28, 39]. For quantification of content or active ingredient, the coefficient must be greater than or equal to 0.99 [39].

As shown in Table 2, correlation coefficients for calibration curve of each peak-component are greater than 0.99. LOD and LOQ for peaks-components (1), (2), (3), and (4) were determined, being 53.73 and 162.83, 60.00 and 181.80, 44.27 and 134.15, and 101.47 and 307.49 *μ*g/mL, respectively. Da Silva et al. quantified simultaneously quercetin and rosmarinic acid in sage and savoury (*Salvia *sp. and* Satureja montana*, resp.) from a calibration curve with standard compounds. LOD and LOQ were found to be 20 and 80 *μ*g/mL for rosmarinic acid, respectively. For quercetin, they were 30 and 90 *μ*g/mL, respectively [40]. In our study, higher values have been determined due to LOD and LOQ values and have been established with respect to the concentration of total HFL.

Intra- and interday precision of the chromatographic method were determined analyzing three concentrations with six replicates each one, during a single day and on three different days; in order to obtain their RSD, results of these parameters are shown in Table 1. Intra- and interday variations were around 9.34% and 10.51%, respectively. Ying et al. used* Angelica sinensis*, a famous traditional Chinese medicinal herb. Many kinds of compounds have been isolated and identified from the plant. They validated a method for the simultaneous quantification of six active compounds present in* A. sinensis* (ferulic acid, senkyunolide I, senkyunolide H, coniferyl ferulate, Z/E-ligustilide, and Z/E-butylidenephthalide) from a mixture of standards. The precision of the quantification method had an intraday RSD below 2.43% and interday below 5.00% [41].

### 3.3. Preparation and Characterization of the Hexane Fraction from* L. frutescens* Incorporated in Polymeric Nanoparticles

**Figure 3:**
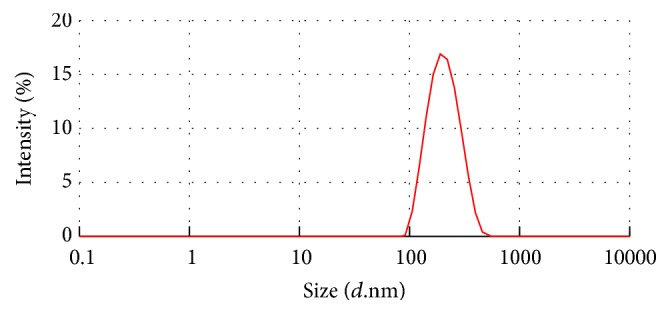
Size distribution of HFL loaded NP measured by the DLS technique.

**Figure 4:**
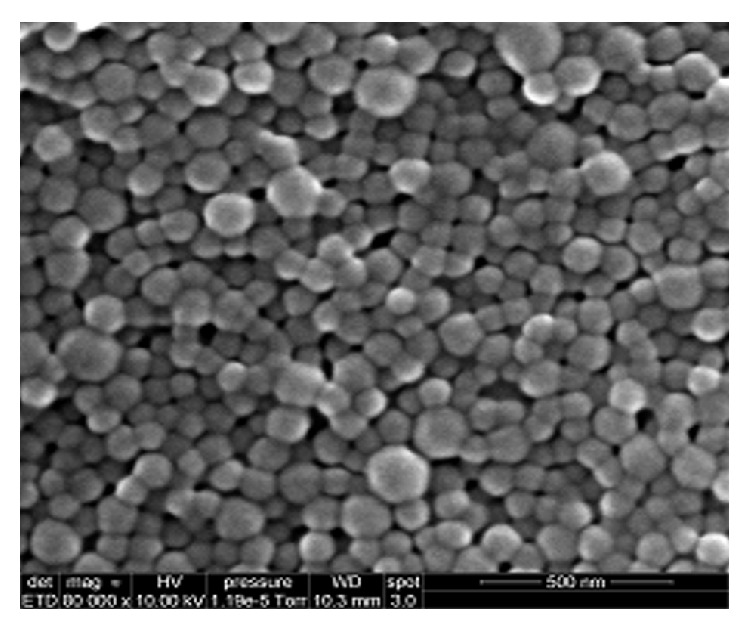
SEM image of PLA nanoparticles prepared by the nanoprecipitation method (scale bar represents 500 nm).

Nanoprecipitation technique described by Fessi et al. was used for NP preparation. The size of obtained NP formulation was 189.70 ± 3.80 nm with homogeneous distribution (Figure 3). SEM was used to visualize the morphology. Particles were evaluated on the basis of shape and presence of interparticulate bridging. Under SEM observation, the submicron particles produced had spherical shapes and showed a homogenous particle size distribution (Figure 4). After that, lyophilized pellet of HFL loaded NP was dissolved in acetonitrile/methanol and solutions were analyzed by HPLC. The area under the curve obtained from each peak-component was replaced in its regression equation (Table 1), in order to obtain the concentration of four peak-components in NP. Consequently, equations (2) were used to determine %  *L* and % EE of each peak-component (Table 2). The encapsulation of the peaks-components in NP was 44, 74, 86, and 97% of peaks (1), (2), (3), and (4), respectively. Based on the separation of peaks by HPLC method, first peak is the less hydrophobic, in NP representing the lowest encapsulation. The most hydrophobic peak in NP represents the highest encapsulation. Studies have reported that nanoprecipitation method is highly favorable in the encapsulation of the hydrophobic compounds [42]. Dalpiaz et al. mentioned that, compared PLGA with PLA, PLA has a lower hydrophilicity [43]. In this study, the method of NP preparation as well as the NP-formed polymer permits higher encapsulation percentage of the hydrophobic peaks-components.

## 4. Conclusions

*L. frutescens *is a plant with potential use. Chromatographic profile is part of characterization of extracts or fractions obtained from plants. In this study, we developed a chromatographic method to obtain the chromatographic profile of hexane fraction of* L. frutescens* leaves. The chromatographic method was validated based on the presence of the peaks-components in HFL, in order to quantify them in NP. HFL loaded NP suspension around 190 nm was obtained. NP-formed polymer and nanoprecipitation method were favorable to encapsulate the hydrophobic components of* L. frutescens* extract.

## Figures and Tables

**Table 1:** Validation parameters for the four peaks-components in hexane fraction from *Leucophyllum frutescens *leaves.

Peak-component	Regression equation	Correlation coefficient (*r*)	LOD (*μ*g/mL)	LOQ (*μ*g/mL)	Intraday precision (RSD, %)	Interday precision (RSD, %)
(1)	*y* = 5.5199*x* − 22.689	0.99	53.73	162.83	4.10	6.08
(2)	*y* = 3.3057*x* − 23.667	0.99	60.00	181.81	6.93	6.41
(3)	*y* = 11.692*x* − 209.84	0.99	44.27	134.15	7.30	7.28
(4)	*y* = 4.8221*x* − 74.844	0.99	101.47	307.49	9.34	10.51

**Table 2:** Characterization of hexane fraction from *L. frutescens *leaves loaded biodegradable polymeric nanoparticles.

Size (nm)	PDI	Quantification		
		Peak-component	% *L*	% EE
189.7 ± 3.81	0.138 ± 0.026	(1)	4.01 ± 0.48	44.23 ± 5.35
		(2)	6.74 ± 0.64	74.34 ± 6.97
		(3)	7.87 ± 0.78	86.78 ± 8.62
		(4)	8.81 ± 0.34	97.03 ± 3.44

Mean ± SD (*n* = 3).
